# The physical performance of workers on offshore wind energy platforms: is pre-employment fitness testing necessary and fair?

**DOI:** 10.1007/s00420-018-1385-5

**Published:** 2018-12-01

**Authors:** Alexandra M. Preisser, Rosalie V. McDonough, Volker Harth

**Affiliations:** Institute for Occupational and Maritime Medicine (ZfAM), University Medical Center Hamburg-Eppendorf, Seewartenstrasse 10, 20459 Hamburg, Germany

**Keywords:** Fitness to work, Aptitude test, Spiroergometry, Oxygen uptake, Heart rate, Offshore, Wind energy

## Abstract

**Purpose:**

Workers on offshore wind turbine installations face a variety of physical and psychological challenges. To prevent potentially dangerous situations or incidents, guidelines for the physical aptitude testing of offshore employees in Germany and other European countries have been developed. However, these criteria have not been previously empirically tested for validity. Although an important component of occupational health and safety, such aptitude testing should not lead to the unjustified exclusion of potential employees.

**Methods:**

Heart rate (HR) and oxygen consumption ($\dot {V}{{\text{O}}_2}$) measurements of 23 male offshore employees and trainers were taken during typical field activities, within the framework of mandatory training exercises. These were evaluated in relation to the individual maximum values of the subjects, determined by cycle spiroergometry.

**Results:**

For the training modules, average HR and $\dot {V}{{\text{O}}_2}$ values of approximately 40% and 33–48% of the maximum values, respectively, were found. Furthermore, 65% of the participants achieved average HR values that exceeded 30% of their individual heart rate reserve and 45% had $\dot {V}{{\text{O}}_2}$ values above 35% of their individual $\dot {V}{{\text{O}}_{2,\text{max} }}$.

**Conclusion:**

Our preliminary results show that offshore work is a form of heavy physical labor, thereby justifying the criteria put forth in the various fitness to work guidelines. We propose that more in-depth investigations should be performed, incorporating task-specific fitness testing as well as higher level aspects of work safety and security, including effective communication skills and teamwork. We also recommend a re-evaluation of the current limits for physical work provided in the literature. The results of such studies could then be applied to other aptitude tests, thereby strengthening the evidence for such measures.

## Introduction

The last 2 decades have brought major technological advances and a significant increase in the industrial use of renewable energy sources. The wind energy sector, for example, has seen particular growth in Europe and China (GWO 2013). This evolution requires an appropriate assessment of the new and changing challenges to health and safety at work. In 2013, EU-OSHA ascertained significant skill gaps in this workforce. Many of the current recommendations on risk assessment, accident prevention, and physical requirements for work have simply been borrowed from related industries, such as the offshore oil and gas industry. Currently, only few standardized training programs specific to the offshore wind energy industry are available (EU-OSHA 2013).

The offshore workplace is dangerous. Employees must be able to perform heavy manual labor, including windlass work and frequent climbing of ladders and stairs (e.g., for 30 min continuous, usually several times a day). Part of the work must be performed at great heights and under often rapidly changing weather conditions. Exposure to multiple physical stressors, including extreme temperatures, continuous noise and vibrations, and a decrease in sleep quality are generally unavoidable (DGAUM 2015; Velasco Garrido 2018a, b). These physical stressors are further compounded by the psychological pressures of being on strenuous 12 h, 14 days on/14 days off work shifts, with limited privacy in the often cramped, shared living quarters, and long absences from home (Parkes 2010; Mette et al. 2018). The consequences of such potentially high-risk situations can be grave and have led to the publication of multiple guidelines to ensure workers’ safety, not only through thorough risk assessment of the workplace, but also through evaluation of the physical and psychological suitability of each employee.

A concept of fitness to work has to be built around the central question: “Can this person do the assigned tasks safely and repeatedly without foreseeable risk to their health and safety or that of their colleagues, third parties and company assets?” (IPIECA and IOGP 2011). Evaluations must consider “direct risks” (i.e., those of the employees and the working conditions themselves) and “indirect risks” (i.e., those that arise due to logistical challenges). The employee must be both “fit for task” and “fit for location” (IPIECA and IOGP 2011). This is especially true for the offshore environment, where medical facilities are often very limited and first-aid measures are the responsibility of the colleagues. In such circumstances, physical restrictions can result in dangerous situations, not only for the injured party, but also for the colleagues and the installations. The purpose of aptitude testing, therefore, should be twofold: it should ensure that employees are able to cope with extreme loads (e.g., ladder climbing and boat landing), as well as manage dangerous situations safely (e.g., rescue by colleagues, firefighting). In this way, danger to the employees themselves, their colleagues, and the platforms should be minimized.

Despite the clear benefit aptitude testing can provide in terms of risk reduction, it also necessarily leads to the exclusion of employees from certain forms of work. This, combined with the lack of evidence or empirical data supporting the validity of the methods used to make assessments of fitness to work, is problematic (Serra et al. 2007; de Kort et al. 1992; de Kort and van Dijk 1997).

Over the past few years, various European guidelines for aptitude testing for work in the offshore environment have been published. Germany, Norway, the UK, and the Netherlands, for example, have each produced a set of preventive measures, including medical or health standards, that employees are to meet before they can be cleared for work offshore (DGAUM/AWMF 2015; NOGEPA 2017; Norwegian Directorate of Health 2015; renewable UK 2013; Taylor 2008). This type of aptitude testing is also seen in other occupational fields, such as firefighting, the military, and law enforcement (Hauschild et al. 2017).

Often, assessments of physical fitness are performed using cardiopulmonary exercise testing by cycle ergometry or with the Chester Step Test (Preisser et al. 2016a). Ensuring the highest level of safety for the employees and their work environment, while also avoiding unnecessary or unfair exclusion, should be a major objective of fitness to work guidelines. It was our goal, therefore, to gain insight into the actual level of physical strain individual employees in the offshore wind energy industry are subjected to during their regular tasks. Direct on-site (offshore) measurements, however, are technically, organizationally, and legally complex. We, therefore, alternatively chose the mandatory (onshore) Global Wind Organization (GWO) safety training modules for our investigation of individual heart rate (HR) and oxygen consumption ($\dot {V}{{\text{O}}_2}$) levels. Although carried out onshore without the added burden of the aforementioned physical and environmental stressors, the practical exercises performed in these sessions are characteristic of the offshore workplace. Furthermore, prior to their first offshore shift and at subsequent regular intervals, every employee is required to complete these trainings.

With this study, we want to verify whether the performance of each participant during the safety trainings is comparable to the individual maximum performance achieved during cycle ergometry, and, as a result, whether this form of exercise testing is a justified and fair aptitude test for work on offshore wind platforms. Recommendations for pre-existing conditions and mental health are also included in the fitness to work guidelines and, although important, they are beyond the scope of this article. The results are presented within the context of the requirements put forth by the guidelines of the German Society for Occupational and Environmental Medicine (DGAUM), published via the Association of the Scientific Medical Societies in Germany (AWMF) (DGAUM 2015), and the British Organisation renewableUK, which released a subsequent guideline specific to the wind energy sector and its risks (renewable UK 2013; Preisser et al. 2016a).

## Methods

During the safety training modules, we were able to recruit 29 participants for our study, only 1 of whom was female. Due to the differing gender reference values for performance, etc., her data were not included in further analyses. Furthermore, because of various organizational circumstances, we were unable to perform cycle cardiopulmonary exercise testing (CPX) or cycle ergometry on all subjects, resulting in a final collective of 23 male subjects. The measurements were taken during the GWO-specified modules such as Working at Heights, Sea Survival, and Fire Awareness, as these reflect the requirements of the regular offshore work most accurately. Measurements were taken from September to November 2016 at the OffTEC Base GmbH & Co. KG in Enge-Sande, Schleswig–Holstein, Germany. Types of training activities and weather conditions of the outdoor and simulated sea survival modules were recorded. Participation was done on a volunteer basis; no pre-selection on the part of the investigators was performed. At the beginning of each of the training modules, participants were informed of the study purpose and objectives, and written consent was obtained.

Prior to exercise testing and field measurements, participants were required to complete a thorough questionnaire concerning their medical history, to detect any prior illnesses and/or risk factors that would have led to exclusion from the study. Spirometry and CPX testing were completed by 16 of the participants, while 7 underwent cycle ergometry without pulmonary data. The forced 1-s and vital capacities (FEV_1_, FVC, and FEV_1_/FVC values) of each consenting individual were determined with spirometry, according to current guidelines (Pellegrino et al. 2005; Criée et al. 2015).

CPX was performed using a cycle ergometer (Speedbike S10.9, Sportsline, Germany) and in accordance with current recommendations (Meyer et al. 2013). During testing $\dot {V}{{\text{O}}_2}$, carbon dioxide production ($\dot {V}{\text{C}}{{\text{O}}_2}$), oxygen saturation (pulse oximetry, SpO_2_), and HR were measured continuously with a pulse belt (Oxycon Mobile by JAEGER^™^/CareFusion, Hoechberg, Germany). Prior to each testing period, the equipment was volume and gas calibrated. A previously defined continuous step protocol was used in all cases: following an initial 1-min period of rest and a 1-min warm-up at 75 W (W), the load was increased by 25 W per minute, until the subjects could no longer maintain the required crank frequency of approximately 60 rpm. The determined ventilatory threshold (VT) corresponds to VT_1_, the point at which blood lactate begins to accumulate and breath frequency increases, in an effort to blow off the higher levels of CO_2_ being produced to buffer acid metabolites. It can be calculated using the V-slope method, i.e., the first disproportionate increase in $\dot {V}{\text{C}}{{\text{O}}_2}$ relative to $\dot {V}{{\text{O}}_2}$ (Schneider et al. 1993; Westhoff et al. 2013). Because offshore employees participate in a thorough physical examination prior to their start of employment, many of the subjects had already performed cycle ergometry. For those subjects who did not undergo CPX “on site”, written consent was obtained to gain access to these test results. Although there were slight variations among the selected protocols, the testing conditions were similar to ours (room temperature, time of day, etc.). In this manner, four additional datasets were obtained for a total of 23 men with exercise testing.

Field HR measurements during the various training modules were taken using HR monitor watch-belt systems (T31 coded transmitter, Polar Electro, Buettelborn, Germany). The activity of each individual was logged for later analysis. A minimum activity period of 2 min was set to account for potential delays in change of HR or recording by the equipment. Long periods of rest (e.g., during instruction, lunch breaks) were not included in the analysis.

For HR and $\dot {V}{{\text{O}}_2}$ measurements, both absolute values and values relative to each individual’s maximum (%HR_max_ and %$\dot {V}{{\text{O}}_{2,\text{max} }}$) were calculated and, where possible, values at the VT (%HR_VT_ and ${\text{\% }}\dot {V}{{\text{O}}_{2,{\text{VT}}}}$). HR_work_ was defined as the difference between the heart rate measured during the trainings and HR_rest_ (Sammito et al. 2015). So-called ‘reserve values’ (i.e., the difference between maximal and minimal measurements) for HR (%HR_R_) and $\dot {V}{{\text{O}}_2}$ (${\text{\% }}\dot {V}{{\text{O}}_{2,{\text{R}}}}$) were defined by the following equations: (HR_work_)/(HR_max_ − HR_rest_) × 100% and ($\dot {V}{{\text{O}}_{2,{\text{training}}}}$ − $\dot {V}{{\text{O}}_{2,{\text{rest}}}}$)/($\dot {V}{{\text{O}}_{2,\text{max} }}$ − $\dot {V}{{\text{O}}_{2,{\text{rest}}}}$) × 100%, respectively. HR and $\dot {V}{{\text{O}}_2}$ at rest were taken as the minimum HR during field measurements (including rest periods) or as the $\dot {V}{{\text{O}}_2}$ preceding CPX, respectively. The results are presented as mean and range.

Due to logistical factors, direct oxygen consumption measurements during field exercises were not possible (time constraints, interference with personal protective equipment (PPE), etc.). As a result, linear regression equations from on-site CPX testing between HR and $\dot {V}{{\text{O}}_2}$ were obtained; the correlation coefficients (*R*) and corresponding *p* values between the individual values of HR and $\dot {V}{{\text{O}}_2}$ were calculated for each of the 16 participants. An average *R* value was then calculated and presented as mean and range. The average $\dot {V}{{\text{O}}_2}$ during the training modules was interpolated from there (Preisser et al. 2016b; Swain et al. 1998). Furthermore, the correlation coefficient (*R*) between the *P*_max_ and $\dot {V}{{\text{O}}_{2,\text{max} }}$ values of the 16 subjects was calculated. Because of the small observation sample size of the different training modules, comparative tests (e.g., *t* test) were not performed.

The participants provided their written informed consent to participate in this study. The study was approved by the Ethics Committee of the Hamburg Medical Association (register number PV5318).

## Results

### Study population characteristics

The majority of the 23 male participants worked in the area of maintenance and repair, although the sample also included people who spend little time offshore, but nevertheless must also complete safety training and undergo aptitude testing. We further included two trainers of the offshore training modules. Due to the young age of the offshore wind energy sector, concrete data on the current working population are rare (Velasco Garrido 2018a; BWE 2015; Kubsova and Felchner 2015). Our collective, however, appears to be similar in age and sex for employees in the offshore wind industry in Germany (Table 1). The group was generally relatively young, with a mean age of 35 years (range 19–68). The mean BMI was 25 kg/m^2^ (range 19.3–33), putting our cohort on the boundary between normal and pre-obesity (Table 1). None of the subjects reported cardiovascular disease in the history and none were under the influence of HR-modifying medications. At the time of study, 30% were active smokers (8.7% < 10/day, 13% 10–20/day, 8.7% > 20/day). All participants had normal spirometry values (data not shown).

**Table 1:** Study population

*n*	Age (years)	Height (cm)	Weight (kg)	BMI (kg/m^2^)
23	35 (19–68)	182 (172–197)	85 (63–110)	25 (19.3–33)

Averages represent values from 23 male participants. Data presented as mean (range)

*BMI* body mass index

### Specifics of the training modules

The training modules such as Working at Heights and Sea Survival each spanned a period of at least 1 day, beginning at approximately 8 a.m. and finishing at approximately 4 p.m., with an hour lunch break and multiple shorter breaks in between. Fire Awareness took place in the afternoons and was approximately 4 h in duration. Working at Heights was performed on two consecutive days. On the first day, for example, the participants were required to carry out rescue situations in wind turbines, using the appropriate safety and rescue devices, and anchor points. They also needed to demonstrate correct behavior on ladders while wearing PPE. On the second day, evacuation exercises from a mock turbine (height 18 m) in full PPE were performed. In addition, the participants discussed and practised strategies to minimize suspension trauma (GWO 2013). The observed Sea Survival units consisted of safe transfer exercises from vessel to dock and vessel to foundation, demonstration of individual and collective survival techniques, and rescuing and first aid of a “man overboard” (GWO 2013). In the Fire Awareness module, participants were asked to demonstrate knowledge of behavior in case of fire, as well as the proper practical application of fire extinguishing equipment (GWO 2013). Activities of modules that were carried out outdoors (i.e., Fire Awareness and Working at Heights) were done at temperatures ranging from 5 to 13 °C, with clear skies and windspeeds no greater than 17 km/h on any given day.

### Results of cycle ergometry and CPX testing

The average max power or load (*P*_max_) for all 23 participants was 242.4 W (range 175–300), or 2.9 W/kg bodyweight (range 1.8–4.0) (Table 2). For the 16 participants who underwent CPX, the $\dot {V}{{\text{O}}_2}$ and determined VT are depicted in Table 3. A positive correlation coefficient (*R*) of 0.79 was observed between the values of *P*_max_ and $\dot {V}{{\text{O}}_{2,\text{max} }}$. In this study, HR_max_ showed a weak negative correlation with age (*R* = − 0.29); however, the mean HR_max_ and $\dot {V}{{\text{O}}_{2,\text{max} }}$ values of the groups were close or equal to the age-predicted values (90.4% (range 71.2–104.9%, *n* = 23) and 100.1% (range 63.8–132.8%, *n* = 16), respectively) (Sammito et al. 2015; Gläser et al. 2013), see Table 4. Furthermore, across all measurements, a strong positive linear relationship was observed between HR and $\dot {V}{{\text{O}}_2}$ values during CPX testing (average *R* = 0.94, range 0.86–0.98, all *p* values < 0.001). This, along with evidence from the literature, justified using the thus derived linear regression equations to calculate $\dot {V}{{\text{O}}_2}$ values during training (Preisser et al. 2016b; Swain et al. 1998). It was also determined that the average $\dot {V}{{\text{O}}_2}$ value at the VT was 69.9% of the average $\dot {V}{{\text{O}}_{2,\text{max} }}$ (range 63.8–90.8%, Table 3). In other words, participants who exceeded their individual VT values during training were working at or above approximately 70% of their $\dot {V}{{\text{O}}_{2,\text{max} }}$. The average weight-adjusted maximum oxygen consumption was 34.5 ml/kg/min (range 18.1–50.7 ml/kg/min), calculated for comparability with the renewableUK guidelines. The loads achieved at a HR of 150 bpm were calculated according to the German guideline (Table 4).

**Table 2:** Results of exercise testing (CPX or cycle ergometry)

	*n*	*P*_max_ (W)	*P*_max_ (W)/BW (kg)	HR_max_ (bpm)
All	23	242.4 (175–300)	2.9 (1.8–4.0)	167 (134–186)
CPX	16	243.8 (175–300)	2.9 (1.8–3.8)	165.5 (134–185)
Ergometry	7	239.3 (200–300)	3.1 (2.3–4.0)	167 (139–186)

Data are presented as mean (range)

*P* power output, *HR*_*max*_ maximal heart rate, *BW* body weight, *CPX* cardiopulmonary exercise testing

**Table 3:** Results of cardiopulmonary exercise testing (*n* = 16) with oxygen consumption ($\dot {V}{{\text{O}}_2}$) and determined ventilatory threshold (VT), also related to maximal values (%*P*_max_, %$\dot {V}{{\text{O}}_{2,\text{max} }}$, %HR_max_)

*n*	HR_VT_ (bpm)	*P*_VT_ (W)	$\dot {V}{{\text{O}}_{2,{\text{VT}}}}$ (ml/min)	%HR_max_	%*P*_max_	%$\dot {V}{{\text{O}}_{2,\text{max} }}$
16	135.1 (112–161)	171.9 (125–225)	2956.6 (1992–3678)	82.2 (69.1–94.7)	70 (54.5–90)	69.9 (63.8–90.8)

Data presented as mean (range)

**Table 4:** Individual HR and $\dot {V}{{\text{O}}_2}$ results, and their evaluation regarding the minimum fitness requirements in the DGAUM/AWMF and renewableUK guidelines

Subject	BMI (kg/m^2^)	CPX	Cycle Ergometry	%HR_max_, pred	%$\dot {V}{{\text{O}}_{2,\text{max} }}$, pred	PWC150 (W/kg BW at 150 bpm)	DGAUM/AWMF	$\dot {V}{{\text{O}}_{2,\text{max} }}$/kg BW	Renewable UK
1	21.8		X	99.3		2.3	Yes		
2	22.7		X	97.8		3.2	Yes		
3	33.5		X	87.7		2.3	Yes		
4	26.0	X		85.8	104.9	2.7	Yes	36.4	Yes
5	29.2	X		89.1	68.6	1.8	No	18.1	No
6	26.3	X		85.0	89.1	2.2	Yes	32.1	No
7	21.2	X		95.2	63.8	1.7	No	27.9	No
8	24.3	X		95.3	95.6	2.4	Yes	40.9	Yes
9	20.6		X	88.5		2.2	Yes		
10	21.3		X	92.2		1.8	No		
11	29.4	X		92.4	102.5	2.6	Yes	29.0	No
12	24.5	X		100.6	73.8	1.2	No	27.2	No
13	24.5		X	82.3		3.7	Yes		
14	24.5	X		96.6	99.9	2.7	Yes	38.2	Yes
15	26.0	X		104.9	108.3	1.7	No	28.2	No
16	25.4	X		95.5	94.9	2.3	Yes	33.0	No
17	33.1	X		82.6	142.8	2.6	Yes	35.3	Yes
18	26.9	X		83.1	110.1	2.7	Yes	35.8	Yes
19	28.2		X	74.2		2.8	Yes		
20	28.8	X		71.2	105.4	2.4	Yes	34.2	No
21	21.0	X		97.7	94.8	3.1	Yes	39.9	Yes
22	22.3	X		91.3	132.3	3.4	Yes	50.7	Yes
23	23.5	X		90.5	115.4	2.6	Yes	45.1	Yes

DGAUM/AWMF requires a minimum of 2.1 W/kg at a HR of 150 bpm, while renewableUK recommends a minimum $\dot {V}{{\text{O}}_{2,\text{max} }}$ of 35 ml/kg/min

### HR field measurements

The results from the HR field measurements from the 23 subjects can be seen in Table 5, grouped according to training module. The varying population sizes are a result of the fact that some study subjects completed multiple training sessions over the course of our investigation (i.e., participation in both Working at Heights and Fire Awareness, Table 6). The maximum absolute value of 205 bpm was observed during Working at Heights (ladder climbing). The highest average HR during the trainings was observed in the Fire Awareness group (113.2 bpm, range 78–154) and the lowest in Sea Survival (105 bpm, range 85–169). In all training modules, the participants were on average working at roughly 65% their HR_max_. Furthermore, the groups did not differ significantly with respect to values relative to their corresponding maxima (%HR_max_) and those at the VT (%HR_VT_). Participants across all training modules achieved average HR_work_ levels that corresponded to approximately 40% HR_R_, see Table 5.

**Table 5:** Average HR_work_ and maximal heart rate during trainings

Module	${n^{\text{a}}}$	Active time (mins)	HR_max,work_ (bpm)		HR_work_ (bpm)		HR_training_%HR_max_^b^		HR_work_%HR_R_^c^
Fire awareness	10	10.1 (7–20)	132.8 (91–176)		43.0 (20–96)		67.2 (42.4–86.3)		43.9 (18.5–75.6)
Sea survival	8	16.25 (5–24)	123.3 (103–169)		33.1 (16–84)		66.5 (55.9–91.9)		38.2 (20.3–84.9)
Working at heights	13	119.8 (39–215)	151.8 (105–205)		38.2 (20–69)		64.6 (48.1–76.3)		39.4 (19.4–64.5)

	*n* ^d^	HR_training_%HR_VT_^b^							
Fire awareness	4	77.8 (59.4–95.7)							
Sea survival	6	77.7 (63.5–85.0)							
Working at heights	10	80.2 (69.5–97.0)							

Data presented as mean (range)

^a^The difference in total measurements from the *n* in Table 1 is due to the fact that some subjects took part in multiple trainings, see also Table 5

^b^HR during the training modules (HR_training_) is also presented relative to the maximal heart rate (HR_training_%HR_max_) and VT (HR_training_%HR_VT)_, as determined by cycle ergometry or CPX. No significant differences between the training modules

^c^HR_work_ is presented relative to heart rate reserve (HR_work_%HR_R_), as determined by cycle ergometry or CPX

^d^Data from those subjects who underwent CPX

**Table 6:** Participants of the various GWO training modules

Subject	Working at heights	Sea survival	Fire awareness
1			X
2		X	X
3			X
4	X		
5	X		
6	X		
7	X		
8	X		X
9	X		X
10	X		X
11	X		X
12	X		X
13	X		
14	X		
15	X		
16	X		
17		X	
18		X	
19		X	X
20		X	
21		X	
22		X	X
23		X	

*GWO* Global Wind Organization

### Oxygen consumption during training

As described above, we found a strong correlation between HR and $\dot {V}{{\text{O}}_2}$, which allowed us to calculate oxygen consumption during the trainings as derived from the measured HR values. The average $\dot {V}{{\text{O}}_2}$ during Fire Awareness, Working at Heights, and Sea Survival modules was 1404.5 ml/min (18.4 ml/min/kg), 1284.8 ml/min (14.9 ml/min/kg), and 923.6 ml/min (10.5 ml/min/kg), respectively (Table 7). Participants of the Fire Awareness training reached average $\dot {V}{{\text{O}}_2}$ levels of approximately 77% and 48% of their respective average $\dot {V}{{\text{O}}_2}$ values at the VT and relative to $\dot {V}{{\text{O}}_{2,\text{max} }}$. For those who took part in Working at Heights and Sea Survival, values of approximately 50% $\dot {V}{{\text{O}}_2}$ at the VT and 35% $\dot {V}{{\text{O}}_{2,\text{max} }}$ were reached (Table 7). These differences were not significant.

**Table 7:** $\dot {V}{{\text{O}}_2}$ during trainings

Module	${n^{\text{a}}}$	Active time (mins)	$\dot {V}{{\text{O}}_{2,{\text{training}}}}$ (ml/min)^b^	$\dot {V}{{\text{O}}_{2,{\text{training}}}}$ %$\dot {V}{{\text{O}}_{2,\text{max} }}$	$\dot {V}{{\text{O}}_{2,{\text{training}}}}$ %$\dot {V}{{\text{O}}_{2,{\text{VT}}}}$
Fire awareness	4	10.75 (7–20)	1404.5 (567.5–2285.2)	48.0 (22.2–60.9)	77.3 (30.4–112.4)
Sea survival	6	15.5 (5–24)	1284.8 (905.6–1609.1)	37.7 (27.8–44.6)	54.3 (32.9–63.1)
Working at heights	10	100.5 (39–174)	923.6 (378.9–1866.6)	33.4 (9.5–63.2)	48.4 (13.9–91.5)

Data presented as mean (range)

$\dot {V}{O_{2,training}}$ oxygen consumption during trainings, *%*$\dot {V}{O_{2,VT}}$ oxygen consumption during trainings relative to that at the ventilatory threshold

^a^The difference in total measurements from Table 2 is due to the fact that some subjects took part in multiple trainings

^b^Values were calculated from individual heart rate/oxygen consumption regression equations, as determined by CPX. No significant differences were found between the training modules

## Discussion

Physical aptitude testing is well established in a variety of physically demanding professions and is thought to represent an important aspect of occupational health and safety. While the benefits of such a practice may seem readily understandable, the general lack of empirical evidence presents a major problem for the field of occupational medicine. Although developed with employees’ best interest in mind, such preventative measures must also strive to avoid the unjust exclusion of people from certain fields of work, such as the offshore wind industry. Here, we provide a first investigation of the physical strain employees are exposed to in this sector. Because it is difficult to conduct on-site workload surveys at offshore workplaces, we examined individuals during compulsory safety training.

### The study group

The average young age of our study group (35) is similar to that which has been observed for the offshore wind sector. As of 2012, roughly 65% of offshore employees worldwide were under 40 years of age, although there remained a small “core” of experienced workers (e.g., from other similar industries such as oil and gas) over the age of 51 (Willis 2012). Our final collective, consisting of 23 men (data from the 1 female subject were not included in statistical analyses), also reflects the gender distribution in the offshore wind energy industry. In Germany, approximately 19,000 people are employed in the offshore wind energy sector (FMEAE 2015). As of 2015, however, women made up not even 10% of all workers (Kubsova and Felchner 2015).

The average BMI of 25 kg/m^2^ places our study sample on the boundary between normal weight and pre-obesity. BMI, however, does not distinguish between muscles, bones, fat mass, and level of physical fitness. Nevertheless, many individuals in our cohort had a low–normal fitness level (based on CPX and predicted maximum values), suggestive of a lack of training.

On average, however, our cohort was within the acceptable range of physical fitness for their age and sex categories, with a mean maximal load of 2.9 W/kg (Table 4). In the literature, expected maximal values of 2.7 (± 0.4) W/kg are given for men between the ages of 30 and 39 (Prokop and Bachl 1984). Furthermore, our group collectively achieved a mean $\dot {V}{{\text{O}}_{2,\text{max} }}$ that was 100.1% of the predicted average maximal value adjusted for age, sex, height, and weight (Gläser et al. 2013). It should be noted, however, that predicted maximal values depend heavily on the particular equation used. Calculations based on another formula recommended by Hansen et al. (1984), for example, resulted in a $\dot {V}{{\text{O}}_{2,\text{max} }}$ value that was only 93.5% of predicted. In any case, our cohort appeared to generally be at or marginally below the predicted values for both HR_max_ and $\dot {V}{{\text{O}}_{2,\text{max} }}$. When considered individually, many of the participants achieved values that were in fact well below the expected (Table 4).

### Offshore work as a form of heavy physical labor

Offshore work in the wind energy industry is said to be physically taxing (DGAUM 2015; Parkes 2010), this has, however, not yet been critically reviewed. Based on HR analyses, our study shows that 65% of the participants achieved average HR_work_ values that exceeded 30% of their HR_R_, a parameter which characterizes hard work, as described below. Furthermore, the mean HR during all trainings was approximately that of the mean HR at 35% $\dot {V}{{\text{O}}_{2,\text{max} }}$ (110.7 bpm). It can, therefore, be assumed that our subjects performed work at a level approximately 35% of their $\dot {V}{{\text{O}}_{2,\text{max} }}$. According to the literature, “limit” values for acceptable levels of strain at work are anywhere between 33 and 50% $\dot {V}{{\text{O}}_{2,\text{max} }}$ for an 8-h shift (Wilson and Corlett 2005; Evans et al. 1980; Åstrand et al. 2003), depending on the number and length of rest periods built into the schedules. For activities that result in prolonged periods of dynamic work, a standardized work–rest schedule (e.g., 50 min on, 10 min off) is recommended (Åstrand et al. 2003). For occupations that involve manual labor with periods of heavy lifting, extreme temperatures, and work in cramped spaces (such as the offshore wind energy industry), even lower limits of approximately 30% $\dot {V}{{\text{O}}_{2,\text{max} }}$ are recommended. Other authors make their recommendations based on the %HR_R_, where 33% is often seen as the upper limit for an 8-h shift (Ilmarinen et al. 1991; Rodgers et al. 1986). Shorter or longer work periods require higher or lower acceptable limits, respectively. For a 12-h shift, for example, Rodgers et al. (1986) recommend an upper limit of 28% $\dot {V}{{\text{O}}_{2,\text{max} }}$. Knowing whether or not work–rest strategies exist for the offshore workplace would allow for a more accurate comparison to the above-mentioned limits found in the literature. Furthermore, the fact that 57% of the participants in the current study achieved HR values that at some point during the trainings exceeded their HR at the individual VT is at least indicative of the intense physical nature of offshore work (individual data not shown). This was particularly observed during Working at Heights where the climbing of ladders is involved. This represents a level of strain at almost 70% of the average maximal value for our study group (Table 6).

### Comparison to other occupations

The average oxygen consumptions during the training modules of 923.6 ml/min (10.5 ml/min/kg, Working at Heights)–1404.5 ml/min (18.4 ml/min/kg, Fire Awareness) is similar to that of employees in other physically demanding occupations. For example, workers of a municipal sanitation department also reached similar values (averaging 1103 ml/min for a period of 1 h) (Preisser et al. 2016b). In this study, the authors found that refuse collection could be classified as heavy work with a high cardiovascular load, based on similar methods of field HR and $\dot {V}{{\text{O}}_2}$ measurements. Studies done on other physically demanding occupations (e.g., the slaughterhouse, healthcare and metal industries, agricultural workers and laborers) also produced similar results (Wultsch et al. 2012; Brighenti-Zogg et al. 2016). In all cases, however, the mean values presented here (%HR_max_ and %$\dot {V}{{\text{O}}_{2,\text{max} }}$) exceeded those found in the named literature.

### Physical requirements for offshore work

The determination of physical fitness is used in the German and UK guidelines to distinguish whether the subject has the physical ability to work on an offshore wind turbine (DGAUM/AWMF 2015, renewableUK 2013). The criterion for performance in the AWMF and renewableUK guidelines is based on heart rate and oxygen uptake, respectively. When looked at individually, half (8/16) of the participants in our study, with an average weight-adjusted maximum oxygen consumption of 34.5 ml/kg/min, did not achieve the oxygen uptake of 35 ml/kg/min required by the ‘renewableUK’ guideline. As well in our study, 21% of the participants would not have met the criteria for offshore work according to the current German guidelines (2.1 W/kg for men at a HR of 150 bpm) (Table 4).

Taken together, the results of our study show that the average fitness level of the group is at the lower end or below that what is required, despite its average young age. This could be due to a number of reasons, including sedentary lifestyle, smoking, (pre-)obesity, or a general lack of training. Because many individuals were well below their expected values for HR and $\dot {V}{{\text{O}}_{2,\text{max} }}$ (Table 4), had a high-normal BMI, and were active smokers, it is likely a culmination of the above factors. Another reason for the poor performance could be due to the fact that oxygen intake in our case was directly determined from CPX and heart rate, while the renewableUK guideline recommends $\dot {V}{{\text{O}}_{2,\text{max} }}$ determination via the so-called ‘Chester Step Test’ (CST). In the CST, the $\dot {V}{{\text{O}}_{2,\text{max} }}$ is calculated using an HR-based method; in contrast to CPX, there is no direct measurement of $\dot {V}{{\text{O}}_{2,\text{max} }}$ during the CST.

Furthermore, the calculated maximum values used here for comparison are provided as health recommendations for the general population, not for those who are employed in physically strenuous occupations, where one would expect the criteria to be more stringent. Finding a balance between safety and fairness is a challenging task when drafting fitness to work guidelines for these employees. None of the participants in this study reported accidents or illness while offshore; however, most were either new to the industry or had not been in employment for very long. Nevertheless, despite the general lack of accident and illness statistics for this industry, our knowledge of the offshore environment and its dangers reinforces the need for pre-employment fitness testing, to best ensure the safety of individual employees, their colleagues, and the platforms.

The few reports published for offshore oil and gas, and for the wind industry showed that the majority of illnesses were musculoskeletal in nature (Norman et al. 1988; Ponsonby et al. 2009; Thibodaux et al. 2014; Jürgens and Weinrich 2015). Musculoskeletal injuries are accountable for more missed workdays than any other form of illness for all occupations and have long been shown to be related to physical fitness (Rayson 2000). This, along with the results of this study, points to a lack of adequate fitness, which is especially relevant regarding the physically demanding nature of work in the offshore environment.

### Study limitations

Due to logistical constraints, it was not possible to accompany the study participants to their actual offshore workplace. It is important to note, therefore, that, while our results do show high levels of strain for the offshore employees, data were collected during training modules. The tasks performed here, however, are comparable to those performed offshore, albeit simulated under the supervision of professionals. The assumption that this level of physical stress is transferable to work on the real platforms could lead to an underestimation of the actual physical workload because there are no additional safety measures (e.g., presence of trainers) as in the safety training examined here. The harsh conditions observed offshore (e.g., extreme temperature, weather) also have an impact on an individual employee’s performance. A significant amount of energy is needed to maintain body temperature homeostasis, thereby decreasing the working capacity of the employee. Our study was carried out either indoors or at relatively mild temperature and weather conditions.

Due to the combination of time constraints of the training schedules and the complex nature of the examinations, it was not possible to recruit and test a larger number of participants. In addition, while only HR recordings during the performance of actual tasks were included in our calculations (i.e., prolonged periods of rest/breaks were excluded), there were indeed rather long stretches of inactivity between the modules. As a result, an entire day’s worth of recording amounted to anywhere from 1 to 4 h of useable data, which could not be extrapolated to a full 8–12-h workday. Also, despite the good level of correlation between HR and $\dot {V}{{\text{O}}_2}$ seen here and in other studies, it is important to consider that HR is an unspecific strain response. Factors such as the type of activity, psychologically stressful situations, and simultaneous heat or cold exposure can all affect its value (Sammito et al. 2015; Wilson and Corlett 2005). Although these effects are possibly negligible in a physically demanding work setting, it is not uncommon for employees in the offshore industry to be exposed to some or even all of these conditions during their rotations. Direct measurement of oxygen consumption at the offshore workplace would, therefore, be a better way to measure physical burden but based on the authors’ experience to date impractical.

## Conclusion

The high physical demands of the offshore workplace are obvious and are, therefore, reflected in the physical fitness requirements put forth in the various guidelines. CPX or cycle ergometry testing only represent one (albeit fundamental) facet of the requirements of the offshore environment. Fitness must also be evaluated with respect to other aspects of work safety, including the individual ability to assess risk, to communicate effectively and work reliably with colleagues, and to handle unanticipated situations in a skilled and efficient manner. We present only a starting point for future studies, however, and suggest that more in-depth investigations should be performed, both to assess the physical strain experienced by offshore employees and to re-evaluate the current limits for physical work provided in the literature. Once achieved, it may be pertinent to incorporate task-specific fitness testing into evaluations of aptitude, as a means to better assess and prepare employees for their desired place of work. Such abilities cannot be measured in a laboratory or field setting with the use of specific equipment but are nonetheless vital to the smooth and safe functioning of the workplace. Appropriate training concepts must, therefore, also place emphasis on teamwork, for example, by including interactions with experienced colleagues in the modules. This study also demonstrates the need for a review and thorough evaluation of the eligibility criteria and their foundation, as formulated in the current guidelines and recommendations.

## References

[CR1] Åstrand PO, Rodahl K, Dahl HA, Strømme SB (2003). Textbook of work physiology. Physiological bases of exercise.

[CR3] Brighenti-Zogg S, Mundwiler J, Schüpbach U, Dieterle T, Wolfer DP, Leuppi JD (2016). Physical workload and work capacity across occupational groups. PLoS One.

[CR4] BWE Bundesverband Windenergie (2016) Beschäftigte in der Windindustrie. (Stand:04/2018). https://www.wind-energie.de/themen/zahlen-und-fakten/deutschland. Accessed 30 Nov 2018

[CR5] Criée CP, Baur X, Berdel D, Bösch D, Gappa M, Haidl P, Husemann K, Jörres R, Kabitz HJ, Kardos P, Köhler D, Magnussen H, Merget R, Mitfessel H, Nowak D, Ochmann U, Schürmann W, Smith HJ, Sorichter S, Voshaar T, Worth H (2015). Standardization of spirometry: 2015 update. Published by German Atemwegsliga, German Respiratory Society and German Society of Occupational and Environmental Medicine. Pneumologie.

[CR6] de Kort W, van Dijk F (1997). Preventive effectiveness of pre-employment medical assessments. Occup Environ Med.

[CR7] de Kort WL, Uiterweer HW, van Dijk FJ (1992). Agreement on medical fitness for a job. Scand J Work Environ Health.

[CR8] DGAUM Deutsche Gesellschaft für Arbeitsmedizin und Umweltmedizin (2015) S1-Leitlinie 002/43: Arbeitsmedizinische Eignungsuntersuchung für Arbeitnehmer auf Offshore-Windenergieanlagen und anderen Offshore-Installationen. http://www.awmf.org/uploads/tx_szleitlinien/002-043l_S1_Arbeitsmedizinische_Eignungsuntersuchung_Offshore_2015-02.pdf. Accessed 19 Oct 2017

[CR9] EU-OSHA European Agency for Safety and Health at Work (2013) Occupational safety and health in the wind energy sector. European Risk Observatory. https://osha.europa.eu/en/tools-and-publications/occupational-safety-and-health-in-the-wind-energy-sector. Accessed 19 Oct 2017

[CR10] Evans WJ, Winsmann FR, Pandolf KB, Goldman RF (1980). Self-paced hard work comparing men and women. Ergonomics.

[CR11] Federal Ministry for Economic Affairs and Energy (2015) Offshore wind energy—an overview of activities in Germany. Berlin: FMEAE. https://www.bmwi.de/Redaktion/EN/Publikationen/offshore-wind-energy.pdf?__blob=publicationFile&v=3. Accessed 31 May 2018

[CR12] Gläser S, Ittermann T, Schäper C, Obst A, Dörr M, Spielhagen T, Felix SB, Völzke H, Bollmann T, Opitz CF, Warnke C, Koch B, Ewert R (2013). The Study of Health in Pomerania (SHIP) reference values for cardiopulmonary exercise testing. Pneumologie.

[CR13] GWO Global Wind Organization (2013) Basic safety training (BST) (Onshore/Offshore). Version 5. http://www.ewea.org/fileadmin/files/our-activities/policy-issues/health-and-safety/GWO_BST_Standard.pdf. Accessed 19 Oct 2017

[CR14] Hansen JE, Sue DY, Wasserman K (1984). Predicted values for clinical exercise testing. Am Rev Respir Dis.

[CR15] Hauschild VD, DeGroot DW, Hall SM, Grier TL, Deaver KD, Hauret KG, Jones BH (2017). Fitness tests and occupational tasks of military interest: a systematic review of correlations. Occup Environ Med.

[CR16] Ilmarinen J, Louhevaara V, Korhonen O, Nygard CH, Hakola T, Suvanto S (1991). Changes in maximal cardiorespiratory capacity among aging municipal employees. Scand J Work Environ Health.

[CR17] IPIECA and IOGP Global oil and gas industry association for environmental and social issues (IPIECA) and International Association of Oil and Gas Producers (IOGP) (2011) Fitness to work: guidance for company and contractor health, HSE and HR professionals. https://de.scribd.com/document/358970354/Fitness-to-work-pdf. Accessed 19 Oct 2017

[CR18] Jürgens C, Weinrich N (2015) Abschlussbericht—Erarbeitung eines Rettungskettenkonzepts für Unfallverletzte in Offshore-Windenergieanlagen. Berufsgenossenschaftliches Unfallkrankenhaus Hamburg. https://www.bg-klinikum-hamburg.de/fileadmin/Dateien/buk-hamburg/PDF/Content/05_Forschung/ABSCHLUSSBERICHT_ROW_FINAL.pdf. Accessed 19 Oct 2017

[CR19] Kubsova J, Felchner C (2015) Unter Männern. https://www.energie-winde.de/mensch-und-umwelt/details/die-vorkaempferinnen.html. Accessed 20 Oct 2017

[CR20] Mette J, Velasco Garrido M, Preisser AM, Harth V, Mache S (2018). Healthy offshore workforce? A qualitative study on offshore wind employees’ occupational strain, health, and coping. BMC Public Health.

[CR21] Meyer FJ, Borst MM, Buschmann HC, Ewert R, Friedmann-Bette B, Ochmann U, Petermann W, Preisser AM, Rohde D, Ruhle KH, Sorichter S, Stahler G, Westhoff M, Worth H (2013). Exercise testing in respiratory medicine. Pneumologie.

[CR22] NOGEPA Netherlands Oil and Gas Exploration and Production Association (2017) Industry standard no. 11 offshore medical examination. https://www.nogepa.nl/download/standard-11-offshore-medical-examination/?lang=en&wpdmdl=2571. Accessed 30 Nov 2018

[CR23] Norman JN, Ballentine BN, Brebner JA, Brown B, Gauld SJ, Mawdsley J, Roythorne C, Valentine MJ, Wilcock SE (1988). Medical evacuations from offshore structures. Br J Ind Med.

[CR24] Norwegian Directorate of Health (2015) Guidelines to regulations regarding health requirements for persons working on installations in petroleum activities offshore. https://www.fylkesmannen.no/globalassets/fm-rogaland/dokument-fmro/helse-og-sosial/regelverk-og-styringsdokument/is-1879e---engelsk-versjon-2015-1.pdf. Accessed 30 Nov 2018

[CR25] Parkes KR (2010) Offshore working time in relation to performance, health and safety. A review of current practice and evidence. Prepared by the University of Oxford for the Health and Safety Executive (HSE). http://www.hse.gov.uk/research/rrpdf/rr772.pdf. Accessed 19 Oct 2017

[CR26] Pellegrino R, Viegi G, Brusasco V, Crapo RO, Burgos F, Casaburi R, Coates A, van der Grinten CPM, Gustafsson P, Hankinson J, Jensen R, Johnson DC, MacIntyre N, McKay R, Miller MR, Navajas D, Pedersen OF, Wanger J (2005). Interpretative strategies for lung function tests. Eur Respir J.

[CR27] Ponsonby W, Mika F, Irons G (2009). Offshore industry: medical emergency response in the offshore oil and gas industry. Occup Med (Lond).

[CR28] Preisser AM, McDonough RV, Harth V (2016). Fitness to work: a comparison of European guidelines in the offshore wind industry. Int Marit Health.

[CR29] Preisser AM, Zhou L, Velasco Garrido M, Harth V (2016). Measured by the oxygen uptake in the field, the work of refuse collectors is particularly hard work: are the limit values for physical endurance workload too low?. Int Arch Occup Environ Health.

[CR30] Prokop L, Bachl N (1984). Alterssportmedizin.

[CR31] Rayson MP (2000). Fitness for work: the need for conducting a job analysis. Occup Med (Lond).

[CR32] Renewable UK (2013) Medical fitness to work—wind turbines: guidelines for near offshore and land based projects. Health and safety issue 2. https://dave-app.com/wp-content/uploads/2014/06/medical_fitness_to_work_54918.pdf. Accessed 30 Nov 2018

[CR33] Rodgers SH, Kenworth DA, Eggleton EM (1986). Ergonomic design for people at work.

[CR34] Sammito S, Thielmann B, Seibt R, Klussmann A, Weippert M, Böckelmann I (2015). Guideline for the application of heart rate and heart rate variability in occupational medicine and occupational science. ASU Int Ed.

[CR35] Schneider DA, Phillips SE, Stoffolano S (1993). The simplified V-slope method of detecting the gas exchange threshold. Med Sci Sports Exerc.

[CR36] Serra C, Rodriguez MC, Delclos GL, Plana M, Lopez LIG, Benavides FG (2007). Criteria and methods used for the assessment of fitness for work: a systematic review. Occup Environ Med.

[CR37] Swain DP, Leutholtz BC, King ME, Haas LA, Branch JD (1998). Relationship between % heart rate reserve and % V̇O_2_ reserve in treadmill exercise. Med Sci Sports Exerc.

[CR38] Taylor J (2008) Medical aspects of fitness for offshore work: guidance for examining physicians—issue 6. London: Oil and Gas UK. http://oilandgasuk.co.uk/product/medical-aspects-of-fitness-for-offshore-work-guidance-for-examining-physicians-issue-6-cd-rom/. Accessed 20 Oct 2017

[CR39] Thibodaux DP, Bourgeois RM, Loeppke RR, Konicki DL, Hymel PA, Dreger M (2014). Medical evacuations from oil rigs off the Gulf Coast of the United States from 2008 to 2012: reasons and cost implications. J Occup Environ Med.

[CR40] Velasco Garrido M, Mette J, Mache S, Harth V, Preisser AM (2018). A cross-sectional survey of physical strains among offshore wind farm workers in the German exclusive economic zone. BMJ Open.

[CR41] Velasco Garrido M, Mette J, Mache S, Harth V, Preisser AM (2018). Sleep quality of offshore wind farm workers in the German exclusive economic zone—a cross-sectional study. BMJ Open.

[CR42] Westhoff M, Rühle KH, Greiwing A, Schomaker R, Eschenbacher H, Siepmann M, Lehnigk B (2013). Positional paper of the German working group “cardiopulmonary exercise testing” to ventilatory and metabolic (lactate) thresholds. Dtsch Med Wochenschr.

[CR43] Willis B (2012) Building a career in offshore wind. http://www.windpowermonthly.com/article/1168428/building-career-offshore-wind. Accessed 5 May 2017

[CR44] Wilson JR, Corlett N (2005). Evaluation of human work.

[CR45] Wultsch G, Rinnerhofer S, Tschakert G, Hofmann P (2012). Governmental regulations for early retirement by means of energy expenditure cut offs. Scand J Work Environ Health.

